# The impact of rural health insurance on vulnerability to chronic poverty among rural residents in China: analysis using Probit and IVprobit models

**DOI:** 10.3389/fpubh.2024.1481019

**Published:** 2024-10-22

**Authors:** Min Zhang, Mu Wu

**Affiliations:** Center for Studies of Ethnic Groups in Northwest China of Lanzhou University, Lanzhou, China

**Keywords:** rural health insurance, rural residents, vulnerability to poverty, China, poverty governance

## Abstract

**Introduction:**

Poverty is a global problem, and combating it is a major governance issue worldwide. In China, poverty management efforts have shifted from eliminating absolute poverty to managing relative poverty. One of the most important tasks in managing relative poverty in the post-poverty reduction era is to prevent recurring poverty due to illness. Rural health insurance is an important method of preventing and mitigating disease risk and a key part of China’s poverty reduction policy, including among rural residents. However, the mechanism by which basic health insurance alleviates vulnerability to poverty, the causal effect of rural health insurance on vulnerability to poverty, and differences based on having a second health insurance policy and by age and income have not been sufficiently explored. Therefore, this study examined the impact of rural health insurance on vulnerability to long-term poverty among rural residents in China. In addition, this study evaluated the impact of having a second health insurance policy and differences in the effects of rural health insurance by age and income.

**Methods:**

This study used data from the 2014, 2016, 2018, and 2020 China Family Panel Studies. Household-related characteristics, such as finance, education, and healthcare, were retrieved from the household database; while data on healthcare expenditures, healthcare insurance, and personal-related characteristics were retrieved from the adult database. Robustness tests were conducted with Probit models, and endogeneity tests were conducted with IVprobit models.

**Results:**

Vulnerability to poverty was significantly lower among residents with rural health insurance than those without any health insurance coverage, and this difference was observed across age and income groups. These findings were consistently robust and significant after controlling for endogeneity, considering sample selectivity, and eliminating measurement bias in the core variables.

**Conclusion:**

The findings indicate that rural health insurance is an important tool for poverty alleviation. The current results could effectively reduce the economic vulnerability of rural households facing health risks, ensuring broader economic security. Moreover, these findings provide policy references for managing relative poverty in China.

## 1 Introduction

Poverty eradication is a long-term task for China’s economic and social development. Since the reform and opening up, China has implemented a series of poverty reduction strategies and has made unprecedented achievements. By 2020, China had eliminated absolute and regional poverty.^1^ However, as poverty is characterized by complexity, systemicity, and dynamic change, the problems of relative and recurring poverty, particularly due to illness, remain prevalent. Preventing recurring poverty in rural areas has become the new focus of poverty governance. Using health insurance to prevent future risks and establishing a long-term mechanism to address relative poverty are key ways to solidify the results of poverty alleviation and build a firm foundation for poverty eradication in China. Therefore, accurately assessing rural residents’ vulnerability to poverty and analyzing the impact of basic health insurance on vulnerability to poverty and its internal mechanisms are important for consolidating the results of poverty eradication and promoting common prosperity in China.

Rural health insurance is an important part of the social insurance system and is a policy tool for preventing poor health and mitigating disease shocks. Since 2003, when the new rural cooperative medical care system was piloted, China has continued to develop a basic health insurance system for rural areas, with the health insurance system for urban and rural residents as its pillar, supplemented by a system of insurance for major illnesses and medical assistance. This has provided full coverage of the rural population. Since 2010, the coverage rate of rural health insurance in China has significantly increased. According to data released by the National Healthcare Security Administration of China, by 2018, the coverage rate of basic medical insurance nationwide had stabilized at over 95%, achieving almost universal coverage. For example, in 2020, the *per capita* funding for basic medical insurance for rural residents in China was 833 yuan, of which government financial subsidies were at least 550 yuan, and individual contributions amounted to 280 yuan per person per year. During the same period, the *per capita* disposable income of rural residents in China was 17,131 yuan, with health insurance expenditures accounting for about 1.63% of total income. In China, the reimbursement policy for the New Rural Cooperative Medical Insurance varies by region and by the level of medical institutions, typically with a deductible. For example, in Hunan Province (a province in central China), the deductible for hospitalization at township hospitals is 200 yuan, 500 yuan at county-level hospitals, 700 yuan at city-level hospitals, and 1,000 yuan at provincial-level hospitals. In addition to the New Rural Cooperative Medical Insurance provided by the state, rural residents may also benefit from public health insurance, urban employee medical insurance (some rural residents may work in cities), and private health insurance, among others. This study identifies groups with any two or more health insurance policies as “having secondary medical insurance.” According to the descriptive statistics in Table 1, 3.9% of the sample population has secondary health insurance.

**Table 1:** Descriptive statistics.

Variable	Full sample (*N* = 48,712)		Having medical insurance (*n* = 45,490)		Not having medical insurance (*n* = 3,222)	
	Mean	SD	Mean	SD	Mean	SD
Vulnerability	0.392	0.112	0.392	0.111	0.403	0.117
Having medical insurance (Yes = 1, No = 0)	0.934	0.249	1	0	0	0
Having secondary medical insurance (Yes = 1, No = 0)	0.039	0.193	0.041	0.199	0	0.018
Sex (Male = 1, Female = 0)	0.499	0.500	0.501	0.500	0.467	0.499
Age	51.379	14.899	51.477	14.797	49.994	16.216
Family members	4.548	2.126	4.559	2.112	4.39	2.302
Years of education	5.585	4.599	5.597	4.583	5.428	4.821
Income	4.477	4.383	4.489	4.375	4.304	4.498
Net house asset	9.675	24.097	9.602	23.581	10.710	30.457
Debt	0.986	3.95	0.990	3.952	0.932	3.909

Since the 18th National Congress of the Communist Party of China, China has introduced a series of health insurance poverty alleviation initiatives from the central government to the local level. These initiatives provided systematic guarantees to lift the rural poor out of poverty (nearly 10 million households). Therefore, considering China’s experience of escaping absolute poverty, the impact of rural health insurance on vulnerability to poverty must be analyzed. According to data from the Poverty Alleviation Office of the State Council of the People’s Republic of China, poverty due to illness was the primary poverty-causing factor for rural residents as of 2015. People continue to face a high burden of medical expenses, especially in middle- and low-income groups, and the phenomenon of poverty caused by illness and recurring poverty due to catastrophic medical expenses occurs occasionally. One of the most important tasks in combating relative poverty in the post-poverty alleviation era is to prevent recurring poverty due to illness. Health insurance is an important method of preventing and resolving disease risks, and the effect of health insurance participation and medical treatment on poverty due to illness expenses^2^ should be comprehensively examined (1).

Poverty is a common problem worldwide, including in both developed and developing countries. Scholars have examined the association between health insurance coverage, poverty, and government financial support in developed countries, such as the United States and Germany. Empirical evidence of catastrophic and poverty expenditures in Germany’s dual-insurance system has been used to analyze financial risk protection in private health insurance (2–5). Some developing countries, such as Senegal, Congo, India, Pakistan, and Bangladesh, have achieved national health insurance coverage and reduced healthcare expenditures for poor people; however, inequalities in health insurance coverage have been shown (6–10). In Namibia and Ghana, reorienting public expenditure policies on healthcare and education through national health insurance schemes has improved access for the poor (11, 12). Health insurance in China is an effective measure to alleviate household poverty and inequality based on financial status. Policies should introduce comprehensive insurance systems with varied reimbursement expense rates for different groups to alleviate poverty (13–16). Scholars have also explored the link between health insurance and specific diseases, analyzing factors such as the community environment, racial disparities, economic policies, and cultural systems (17–20).

In China, health is a key factor in individuals’ impoverishment, and health insurance can effectively counteract health shocks. Illness reduces farmers’ labor efficiency, supply, and income. Medical expenditures increase farmers’ economic burden. If medical expenditures exceed affordability, they can become catastrophic, causing a vicious circle of poverty and illness. Therefore, studies have examined the impact of health insurance on vulnerability to poverty. Pigou argued that the state’s participation in income distribution through the social insurance system promotes income equalization and helps maximize social welfare, which provides solid theoretical support for poverty reduction through health insurance (21). Liu Yuanli was the first to propose the theory of poverty reduction through health insurance. This was supplemented and improved by Yip and Hsiao, who proposed that health insurance alleviates poverty caused by an illness by compensating for the medical burden, with the amount of compensation negatively correlated with out-of-pocket medical expenditures and positively correlated with the poverty reduction effect (22). Health insurance directly affects poverty by compensating for individual medical consumption and indirectly affects it by improving the individual’s capital stock. It improves people’s health and increases investment in education (23–25). Thus, health insurance effectively mitigates the impact of diseases on individual income, reduces the likelihood of individuals falling into poverty, prevents recurring poverty, and consolidates the results of poverty eradication.

Previous studies have explored poverty governance using basic health insurance. Asfaw and Jütting found that in developing regions, such as Africa, health insurance had the dual effect of increasing healthcare service use and lowering the incidence of poverty (26). Sommers and Oellerich revealed that U.S. Medicare significantly reduced medical out-of-pocket expenditures and alleviated poverty (27). Gu et al. demonstrated that health insurance improved health and alleviate poverty caused by illness among urban residents in China (28). Poverty governance in China should focus on relative poverty in the future as well as the present. Existing studies have used poverty incidence and intensity to measure poverty; however, the problems are complex. Vulnerability to poverty better reflects the risk of individual farmers falling into relative poverty in the future. Zining et al. revealed that health insurance effectively reduced vulnerability to poverty, with a more significant effect among people with poor health (29). Gao and Ding argued that the New Rural Cooperative Major Disease Insurance effectively alleviated long-term poverty in rural areas (30).

However, the mechanism by which basic health insurance alleviates vulnerability to poverty has not been fully and systematically investigated. Moreover, the causal effect of rural health insurance on rural residents’ vulnerability to poverty has not been sufficiently explored. Most existing studies have chosen a single year but have not conducted tracking surveys or continuous analyses. In addition, studies have not analyzed the population with a second health insurance policy and people of different age groups, ignoring the differences and long-term dynamics of health insurance among different population groups. Therefore, this study examined vulnerability to long-term poverty among the rural population in China and explored differences based on having a second health insurance as well as by age and income. This study could help improve the health insurance policy for impoverished people in rural areas and provide an important reference for comprehensively assessing the performance of rural health insurance implementation in China.

## 2 Methods

### 2.1 Data sources

This study used data from the 2014, 2016, 2018, and 2020 China Family Panel Studies (CFPS),^3^ which surveyed social, economic, demographic, educational, and health aspects at the individual, household, and community levels. The CFPS sample covers 25 provinces, municipalities, and autonomous regions in China, with a sample size of 16,000 households, including all household members.

This study used data of rural residents aged 25–85 years. Individuals in this age range are typically married and have health insurance; thus, the population is representative of the situation in China. First, we identified the household characteristics and personal information of the head of the household through the household and adult databases. We retained only the data of rural residents as identified in household registers. Second, we retrieved data on household-related characteristics, such as income, education, and healthcare, from the household database and data on medical expenditures, health insurance, personal characteristics, and number of households from the adult database.^4^ Finally, we matched the household codes with the household head and merged the data samples, resulting in 8,652 households and 48,712 samples.

### 2.2 Variable selection

The explanatory variable in this study was the level of vulnerability to household poverty. Vulnerability to poverty measures the likelihood that a household will fall into poverty in the future, considering not only the current characteristics and economic situation of the household but also a combination of various risk factors that it may face in the future. Vulnerability to poverty comprised three aspects: vulnerability to expected poverty (VEP), vulnerability to low expected utility, and vulnerability to uninsured risk exposure. This study used VEP to measure vulnerability to poverty (31, 32).

This method calculates the period during which a household or individual fell into poverty based on the household or individual characteristics of that period (32, 33). This method was significantly improved by Klasen and Waibel (34). The method regresses income on observable variables and shocks to obtain an expression for future income, which assumes that the logarithm of income follows a normal distribution; thus, the probability that future income will fall below a certain value (usually the poverty line) is referred to as the vulnerability line. The calculation uses the three-stage generalized least squares estimation (feasible generalized least squares) to establish the mean income and income fluctuation model, estimate the logarithm of *per capita* income, and conduct an ordinary least squares regression analysis on the squared residuals after the regression analysis. The specific method for VEP is as the following Equations 1-5:


(1) $$lnIncome_{it}=\alpha {Χ}_{it}+\varepsilon_{i}$$

where ${\mathrm{lnIncome}}_{\mathrm{it}}$ represents the logarithm of family i income level in year t ; ${Χ}_{\mathrm{it}}$ covers family, personal characteristics, and factors of vulnerability to risk shock; and $\varepsilon_{i}$ represents the residual difference of the regression analysis. This variance is then used to estimate the uncertainty of household consumption; that is, the consumption variance of an individual or household. An estimate of this variance can be used to measure the volatility of future consumption levels. To estimate the variance of household income, the heteroscedastic structure weight is constructed as follows:


(2) $$\varepsilon_{i}2=\beta {Χ}_{it}+\xi_{i}$$

where $\varepsilon_{i}2$ is the estimated consumption variance for the 𝑖 household and 𝛽 is the parameter estimated from step 1.

In the second step, the weighted regression analysis of the residual square and logarithm of income is performed again:


(3) $$E\left(lnIncome_{i,t+1}|{Χ}_{it}\right)={Χ}_{it}\hat{\alpha_{FGLS}}$$

(4) $$V\left(lnIncome_{i,t+1}|{Χ}_{it}\right)=\varepsilon_{i}2={Χ}_{it}\hat{\beta_{FGLS}}$$

In the third step, VEP values are calculated by estimating the probability of each household falling into poverty. The VEP represents the probability that a family’s future consumption level will be below the poverty line, which can be calculated using a standard normal distribution function. The logarithm of income is assumed to follow a normal distribution, and the poverty line and vulnerability to poverty threshold are defined, as follows:


(5) $$\hat{VEP_{it}}=\mathrm{Pr}\left(lnIncome_{it}\leq lnPoor\right)=\phi \left[\frac{lnPoor-{Χ}_{it}\hat{\alpha_{FGLS}}}{{Χ}_{it}\hat{\beta_{FGLS}}}\right]$$

The choice of the poverty line directly determines the final analytical results. In 2018, the World Bank identified survival expenditures per person per day of less than $1.9, $3.2, and $5.5 as the extreme, medium-low, and medium-high poverty lines, respectively (35). Based on the 2018 US-Chinese currency exchange rate (1:6.61), the annual poverty lines were RMB 4584, RMB 7720, and 13,270 yuan. During the same period, the poverty line set by China’s Poverty Alleviation and Development Department was RMB 2855; however, according to the State Council Poverty Alleviation Office, most of China’s provinces set local standards higher than the national standard, with 12 provinces setting the poverty line between RMB 4000 and 6,000. According to Zhang and Wan, a higher poverty line corresponds to a higher prediction accuracy; therefore, the medium-low poverty line standard recognized by the World Bank was chosen as the poverty line reference value for this study (36).^5^

The core explanatory variable used in this study was having at least one health insurance policy, expressed as $Medicalinsur_{it}$ .^6^ If the respondent had at least one health insurance policy, the explanatory variable $Medicalinsur_{it}$ was 1, and 0 otherwise. This indicator was identified in the CFPS database and transformed in terms of specific insurance types. Moreover, we examined having a second health insurance policy as the core explanatory variable.

The other control variables were household information, including age, sex, education level, total household income and expenditure in the past 12 months, and medical expenditure of the head of the household; asset and liability indicators, including annual household income, value of the household’s net property, value of productive fixed assets, value of the household’s holdings of financial products, and level of debt; and individual characteristics, including sex, age, marital status, and health. Table 1 presents the descriptive statistics of this study.

### 2.3 Empirical methods

We used the following model to explore the causal effect of health insurance on vulnerability to poverty:


(6) $$Vul_{it}=\beta_{0}+\beta_{1}Medicalinsur_{it}+\beta_{i}{Χ}_{it}+\lambda_{i}+\eta_{t}+e_{it}$$

where $Vul_{it}$ represents household vulnerability to poverty measured under the $3.2/person/day poverty standard; $Medicalinsur_{it}$ represents whether sample i had at least one health insurance in year t ; $\beta_{1}$ is the core regression coefficient; ${Χ}_{it}$ represents the control variable; $\lambda_{i}$ represents the regional fixed effect; $\eta_{t}$ represents the fixed effect of time (year); and $e_{it}$ represents the residual term.

Further, we used a Probit model to estimate Equation 6. The results were used as a robustness test, in which three poverty line probabilities were chosen simultaneously. The first vulnerability to poverty line was 29%; that is, a household was considered vulnerable if its future probability of falling into poverty exceeded 29% (33). The second vulnerability to poverty line was 40%, following Rajadel (37). The third vulnerability to poverty line was 49%, following Chaudhuri et al. (33). If $Vul_{it}\geq Povertyvulnerabilityline$ , household vulnerability to poverty was high, and $Vul_{it}=1$ in the Probit model; otherwise, $Vul_{it}=0$ .

## 3 Results and discussion

### 3.1 Benchmark regression analysis results

Table 2 presents the regression analyses results for vulnerability to poverty based on rural health insurance for the entire sample, including the results using two-way fixed effects and the first vulnerability to poverty line of the Probit model. We controlled for year and province fixed effects and included control variables at the household and head of household levels. The resulting coefficients were all negative and highly significant, indicating that vulnerability to poverty among residents with rural health insurance was significantly lower than among those without any health insurance. Specifically, having health insurance reduced the vulnerability of households by 1.3–1.7% on average.

**Table 2:** The impact of rural health insurance on vulnerability to poverty.

	(1)	(2)	(3)	(4)	(5)	(6)
Variables	Vul	Vul	Vul	Vul	Vep1	Vep1
Medical insurance	−0.0101***	−0.0162***	−0.0169***	−0.0130***	−0.1967***	−0.1799***
	(0.0020)	(0.0019)	(0.0019)	(0.0018)	(0.0245)	(0.0254)
Sex			−0.0127***	−0.0136***		−0.1433***
			(0.0010)	(0.0009)		(0.0128)
Age			0.0009***	0.0007***		0.0093***
			(0.0000)	(0.0000)		(0.0005)
Family members			0.0045***	0.0067***		0.0965***
			(0.0002)	(0.0002)		(0.0033)
Education			−0.0028***	−0.0022***		−0.0272***
			(0.0001)	(0.0001)		(0.0016)
*ln*Income				−0.0083***		−0.1258***
				(0.0002)		(0.0039)
*ln*Net house asset				−0.0039***		−0.0457***
				(0.0002)		(0.0024)
*ln*Debt				0.0040***		0.0352***
				(0.0001)		(0.0015)
Province FE	No	Yes	Yes	Yes	Yes	Yes
Time FE	Yes	Yes	Yes	Yes	Yes	Yes
*N*	48,712	48,712	48,712	48,712	48,712	48,712
*R* ^2^	0.062	0.128	0.174	0.227	-	-

Numbers in parentheses are estimated standard errors. ***p < 0.01. Vul, vulnerability; VEP, vulnerability to expected poverty; FE, fixed effects.

Moreover, we used the Probit model as a comparison and achieved negative and significant results. The Probit model is suitable for regression analyses dealing with dichotomous dependent variables. Although its coefficients cannot be directly interpreted in terms of economic significance, their direction and significance provide important validation. The results of the Probit model demonstrated that rural health insurance had a significant negative effect on vulnerability to poverty, supporting the findings of the fixed-effects model.

Rural health insurance significantly reduced households’ vulnerability to poverty, even after controlling for year- and area-fixed effects, and when head of the household and household-level control variables were included. Robustness tests further confirmed the reliability of this finding through Probit modeling. These results revealed the important role of rural health insurance in alleviating poverty.

Moreover, households headed by men and those with higher levels of education had relatively lower vulnerability to poverty. This trend was negatively affected by the age of the head of household and increase in household size (i.e., higher probability of recurring poverty). From the household perspective, growth in income and assets helped reduce vulnerability to poverty, whereas increases in household debt were detrimental to the decline in vulnerability to poverty, which was consistent with conventional expectations.

### 3.2 Robustness test results

#### 3.2.1 Endogeneity test

Endogeneity between health insurance and vulnerability to poverty could not be ruled out. The risk of endogeneity arose from omitted variables and bidirectional causal effects. As we could not observe the full range of intra-household characteristics, factors such as social networks and cultural differences may have affected the vulnerability of households to poverty. This could be handled by controlling for the fixed effects for each household; however, such an approach would lose degrees of freedom, and the data in this study did not support this approach. Second, poverty may constrain households from investing in health insurance, while simultaneously increasing the risk of poverty due to limitations in wealth levels. This bidirectional causality can complicate the relationship between health insurance expenditure and vulnerability to poverty, hindering the accurate identification of the causal relationship between the two. Therefore, we selected a two-stage instrumental variable approach to test the potential risk.

The instrumental variable of whether to purchase health insurance was household medical expenditure in the past 12 months. This instrumental variable satisfied the correlation requirement, as a higher level of medical expenditure in the previous year may stimulate the household to emphasize health insurance. This crisis effect may stimulate the household’s demand for insurance to avoid medical expenditure. Medical expenditures due to illnesses in general households are often difficult to advance because illnesses are usually episodic and sudden; thus, medical expenditures satisfied the exogeneity requirement of the instrumental variable. We used the two-stage least squares (2SLS) and IVprobit models for endogeneity testing, and the results are shown in Table 3.

**Table 3:** Endogeneity test.

	(1)	(2)	(3)	(4)
Variables	Vul	Vul	Vep1	Vep1
Medical insurance	−3.4938**	−4.4548*	−4.0520***	−4.0618***
	(1.6699)	(2.5438)	(0.0153)	(0.0133)
Individual control variable	No	Yes	No	Yes
Family control variable	No	Yes	No	Yes
Year FE	Yes	Yes	Yes	Yes
Province FE	Yes	Yes	Yes	Yes
N	48,712	48,712	48,712	48,712
F	16.38	14.41	-	-

Numbers in parentheses are estimated standard errors. *p < 0.1, **p < 0.05, ***p < 0.01. Vul, vulnerability; VEP, vulnerability to expected poverty; FE, fixed effects.

The results of the 2SLS instrumental variables test revealed that the *F*-values of the first stage were above the threshold of 10.28 without and after controlling for the individual and household control variables, demonstrating the reliability of the instrumental variables (38). Based on the regression coefficients in the second stage, the effect of health insurance on reducing vulnerability to poverty remained negatively significant. The absolute value of the coefficients was larger than the results of the baseline regression analysis, indicating that the results of the baseline regression analysis underestimated the potential effect of health insurance on reducing vulnerability to poverty after excluding endogenous risks. For robustness considerations, we validated the results of the two-stage Probit model. Although the IVprobit model could not provide the *F*-value of the first stage, the results indicated the reliability of the instrumental variables. The regression analysis results indicated that the current results were robust under the low poverty line criterion.

#### 3.2.2 Excluding measurement bias in core variables

Considering the measure of vulnerability to poverty of the explanatory variables, the empirical findings may be affected by differences in poverty line criteria. Using the definitions in Subsection 2.3, we simultaneously selected three poverty lines for robustness testing. The results are presented in Table 4.

**Table 4:** Robustness tests for different vulnerability to poverty lines.

	(1)	(2)	(3)	(4)	(5)	(6)
	Vep1	Vep 1	Vep 2	Vep 2	Vep 3	Vep 3
Medical insurance	−0.1967***	−0.1799***	−0.2357***	−0.2095***	−0.2561***	−0.2222***
	(0.0245)	(0.0254)	(0.0245)	(0.0255)	(0.0270)	(0.0279)
Individual control variable	No	Yes	No	Yes	No	Yes
Family control variable	No	Yes	No	Yes	No	Yes
Year FE	Yes	Yes	Yes	Yes	Yes	Yes
Province FE	Yes	Yes	Yes	Yes	Yes	Yes
*N*	48,712	48,712	48,712	48,712	48,712	48,712

Numbers in parentheses are estimated standard errors. ***p < 0.01. VEP, vulnerability to expected poverty; FE, fixed effects.

The results demonstrated that the regression coefficients for health insurance were significantly negative regardless of which standard poverty line was chosen and whether control variables were added, indicating that the main conclusions held after excluding measurement bias in the explanatory variables. Based on the regression coefficients, when the standard of vulnerability to poverty was continuously raised, the impact of health insurance on the vulnerability to poverty of rural residents appeared to gradually increase (the absolute value of the coefficients is increasing). This suggests that under the premise of continuously raising the standard of poverty alleviation, health insurance effectively alleviates the economic burden of rural residents in the face of illness and prevents poverty from occurring or recurring owing to illness.

#### 3.2.3 Robustness test using propensity score matching methodology

This study adopted the propensity score matching method to test the robustness of the impact of health insurance on rural residents’ vulnerability to poverty. We divided the sample into a disposition group with at least one health insurance and control group with no health insurance. We adopted the propensity score matching method to estimate whether rural residents chose to invest in health insurance, and measured the sample match between the disposition group and the control group using the 1:1 proximity matching method. We obtained 2,957 pairs of samples and used Model 6 to verify whether the group with health insurance had a lower vulnerability to poverty than the group without any health insurance. The results of the regression analysis are presented in Table 5.

**Table 5:** Robustness test using propensity score matching.

	(1)	(2)	(3)	(4)	(5)	(6)
	Vul	Vul	Vul	Vep1	Vep2	Vep3
Medical insurance	−0.0108***	−0.0108***	−0.0084***	−0.1001***	−0.1128***	−0.1275***
	(0.0029)	(0.0028)	(0.0027)	(0.0364)	(0.0367)	(0.0417)
Individual control variable	No	Yes	Yes	Yes	Yes	Yes
Family control variable	No	No	Yes	Yes	Yes	Yes
Province FE	Yes	Yes	Yes	Yes	Yes	Yes
Time FE	Yes	Yes	Yes	Yes	Yes	Yes
*N*	5,914	5,914	5,914	5,914	5,914	5,914
*R* ^2^	0.109	0.153	0.209	–	–	–

Numbers in parentheses are estimated standard errors. ***p < 0.01. Vul, vulnerability; VEP, vulnerability to expected poverty; FE, fixed effects.

We included individual and household control variables; the regression analysis results of health insurance were consistently negative and significant, and the coefficients did not change significantly. We used three poverty standards; the regression analysis results of health insurance remained negative and significant, and the coefficients in absolute value increased with the increase in the standard of the poverty line. Based on the above validation sets, we concluded that the benchmark regression analysis results in this study were robust.

### 3.3 Impact of second health insurance on vulnerability to poverty

To further explore the impact of health insurance on rural residents’ vulnerability, we retrieved data on whether respondents had a second health insurance policy. We used the same measure as the first health insurance policy but excluded duplicate samples with two policies of the same type. We estimated regression analyses for samples of different ages using 10 years as the age threshold (i.e., six stages using 10-year intervals for those aged 25–85 years). The results are shown in Table 6.

**Table 6:** Impact of second health insurance.

Age (years)	(1)	(2)	(3)	(4)	(5)
	35–44	45–54	55–64	65–74	75–85
	Vul	Vul	Vul	Vul	Vul
Secondary medical insurance	−0.0126*	−0.0161**	0.0025	−0.0219***	−0.0108*
	(0.0074)	(0.0070)	(0.0062)	(0.0052)	(0.0057)
Individual control variable	Yes	Yes	Yes	Yes	Yes
Family control variable	Yes	Yes	Yes	Yes	Yes
Province FE	Yes	Yes	Yes	Yes	Yes
Time FE	Yes	Yes	Yes	Yes	Yes
*N*	48,712	48,712	48,712	48,712	48,712
*R* ^2^	0.173	0.173	0.172	0.173	0.173

Numbers in parentheses are estimated standard errors. *p < 0.1, **p < 0.05, ***p < 0.01. Vul, vulnerability; FE, fixed effects.

For younger (35–54-years-old) and older (65–74-years-old) respondents, the effect of health insurance on reducing their vulnerability to poverty was the most significant. For the oldest respondents (75–85-years-old), the effect of health insurance on reducing vulnerability to poverty was low. For middle-aged respondents (55–64-years-old), the effect of health insurance on vulnerability to poverty was not significant. Younger people are in a period of high household financial burden and usually face expenses, such as raising children and paying mortgages. Health insurance reduces the burden of medical expenditures, helping them avoid financial hardships when facing health problems. In addition, this age group is often a major participant in the labor market, and health insurance coverage reduces the risk of poverty by helping them stay healthy and reducing the loss of income due to illness. Conversely, people aged 65–74 years gradually enter retirement, with fixed income such as pensions becoming the main source of income. The relative burden of medical expenditures is heavier, and health insurance can effectively alleviate this pressure. Further, the health risk of this age group increases significantly, and the protection provided by health insurance can significantly reduce financial pressure when they fall ill and help them avoid financial difficulties caused by high medical expenses.

Older individuals generally have higher healthcare needs and expenditures, and their out-of-pocket expenses are large despite having health insurance, resulting in a small effect of insurance on poverty reduction. In addition, this age group may have already accumulated wealth or may enjoy other forms of social security, and the marginal role of health insurance in the overall economic security is relatively small. Middle-aged individuals are generally working, have a relatively stable source of income, and are more tolerant of healthcare expenditures. Compared with other groups, they may have more savings and assets, and the protective effect of health insurance is relatively small. In addition, this age group may actively invest in their health before retirement and may have lower medical needs and expenditures, making the impact of insurance less significant.

### 3.4 The impact of health insurance on vulnerability to long-term poverty among rural residents

**Figure 1:**
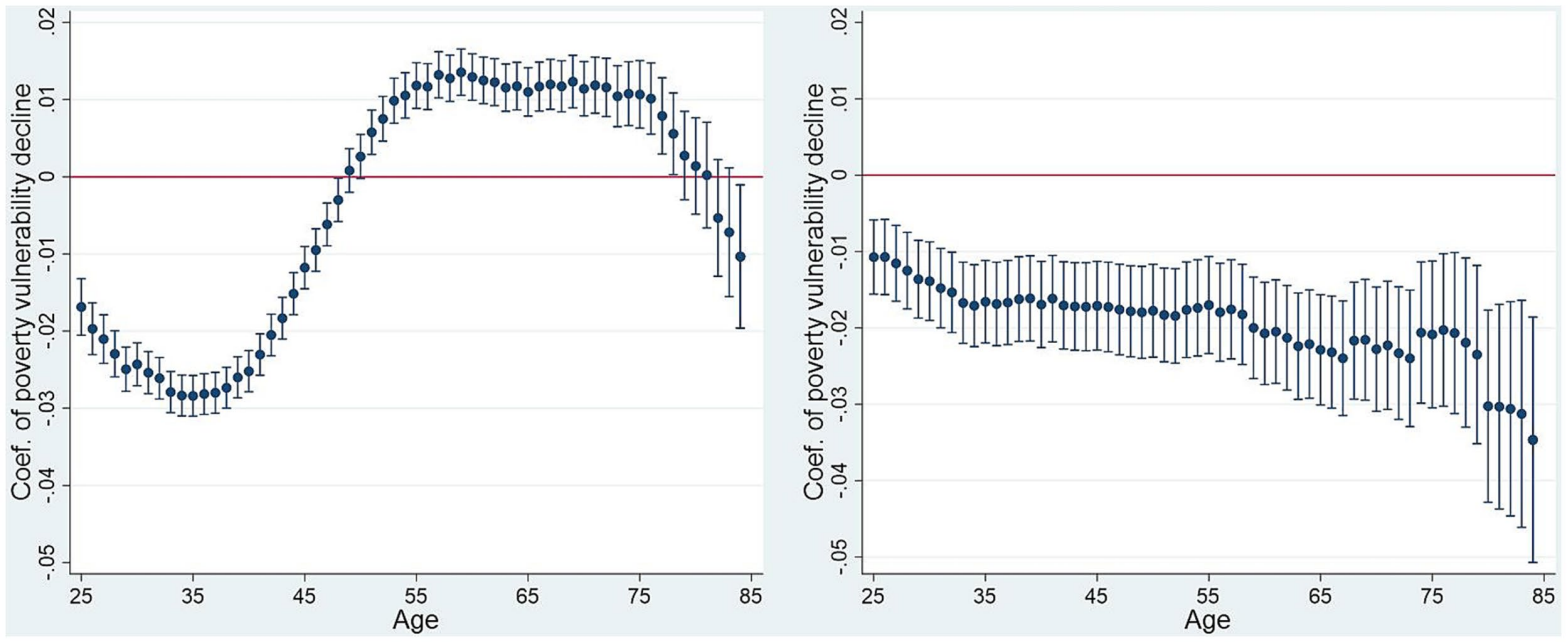
The impact of health insurance on vulnerability to poverty among different age groups.

Moreover, we examined the relationship between age and vulnerability to poverty and visually presented the coefficient changes (Figure 1). Significant differences in health insurance were observed among individuals with one policy by age, which is consistent with the results presented above. For individuals younger than 54 years or older than 78 years, health insurance was effective in reducing the vulnerability to poverty. However, for participants aged 55–78 years may face a financial burden due to healthcare insurance investments, potentially leading them back into poverty. Therefore, we believe that the government should provide more targeted healthcare subsidies rather than applying a uniform approach to all age groups.

Among individuals with a second health insurance policy, health insurance effectively reduced vulnerability to poverty regardless of age. This finding may be closely related to the affluence and health management awareness of the segment itself. Typically, only affluent groups have the ability and willingness to invest in secondary health insurance policies. Households with higher financial strength can spend more on insurance, which provides more comprehensive healthcare coverage and reduces the financial pressure caused by unexpected medical events. The resource advantages of wealthier households help them afford healthcare expenditures, which significantly reduces their vulnerability to poverty. Groups that invest in a second health insurance policy usually have higher awareness of health management. These groups focus on prevention and timely treatment, reducing the probability of serious illnesses and corresponding high medical costs. Such proactive health management behaviors not only help maintain good health but also avoid financial difficulties caused by health problems.

### 3.5 Heterogeneity tests

#### 3.5.1 Heterogeneity analysis using wealth quartiles

Using the income quartile variables provided on the official website of the CFPS, this study examined the population heterogeneity of income classes. The regression analysis results are presented in Table 7.

**Table 7:** Heterogeneity analysis using wealth quartile.

	(1)	(2)	(3)	(4)
	Vul	Vul	Vul	Vul
Medical insurance	−0.0107***	−0.0161***	−0.0099**	−0.0099
	(0.0024)	(0.0032)	(0.0044)	(0.0065)
Individual control variable	Yes	Yes	Yes	Yes
Family control variable	Yes	Yes	Yes	Yes
Province FE	Yes	Yes	Yes	Yes
Time FE	Yes	Yes	Yes	Yes
*n*	17,268	15,111	10,413	5,264
*R* ^2^	0.203	0.229	0.226	0.163

Numbers in parentheses are estimated standard errors. **p < 0.05, ***p < 0.01. Vul, vulnerability; FE, fixed effects.

Although health insurance helped reduce vulnerability to poverty for the poorest 25% of the population, its effect was not significant. This may be because people in this income bracket have extremely weak economic foundations, making it challenging to cope with the financial pressures of medical costs, even if they have health insurance. In addition, the poorest groups may have access to other forms of social security and assistance provided by the government, thereby limiting the marginal effect of health insurance.

Contrastingly, health insurance significantly reduced vulnerability to poverty for the bottom 25–50% of income earners. This group is in a more precarious economic situation but has an income base that allows them to pay for health insurance. Health insurance provides important financial protection against high medical costs associated with illness, which can lead to financial hardship. Moreover, this group may not receive additional government assistance available to the poorest groups, causing the role of health insurance to be particularly critical.

For the more affluent group (top 25–50% of income), the role of health insurance was not significant. This group has higher incomes and savings and can pay for healthcare; thus, health insurance has relatively little impact on their financial situation. The economic security of this group is relatively solid; therefore, the marginal benefits of health insurance are low. For the wealthiest group (top 25% of income), health insurance did not significantly affect vulnerability to poverty. The wealthiest households typically have sufficient financial resources and diversified sources of income to remain economically stable, even when faced with medical expenses. For these households, health insurance is a risk-management tool rather than a tool necessary for financial security.

#### 3.5.2 Heterogeneity analysis using self-health orientation

Further, we conducted a heterogeneity analysis using respondents’ self-health orientation. The results are presented in Table 8. Using the data in the CFPS database, we divided self-health orientation into five categories: unhealthy, generally healthy, relatively healthy, healthy, and very healthy.

**Table 8:** Heterogeneity analysis based on self-health positioning.

	(1)	(2)	(3)	(4)	(5)
	Vul	Vul	Vul	Vul	Vul
Medical insurance	−0.0117***	−0.0227***	−0.0136***	−0.0035	−0.0106**
	(0.0036)	(0.0048)	(0.0033)	(0.0044)	(0.0046)
Individual control variable	Yes	Yes	Yes	Yes	Yes
Family control variable	Yes	Yes	Yes	Yes	Yes
Province FE	Yes	Yes	Yes	Yes	Yes
Time FE	Yes	Yes	Yes	Yes	Yes
*n*	10,423	7,272	16,854	7,781	6,376
*R* ^2^	0.206	0.233	0.223	0.252	0.266

Numbers in parentheses are estimated standard errors. **p < 0.05, ***p < 0.01. Vul, vulnerability; FE, fixed effects.

The results revealed variability in the impact of health insurance based on self-perceived health orientation. The effect of health insurance on vulnerability to poverty was the most significant for respondents with poor self-perceived health. This may be due to their exposure to potential health risks, with health insurance providing timely and effective medical coverage to avoid financial difficulties caused by health problems, thereby reducing vulnerability to poverty.

In addition, health insurance significantly reduced vulnerability to poverty in both unhealthy and relatively healthy groups. For the unhealthy group, the role of health insurance may be more direct and obvious, effectively reducing the economic pressure caused by medical expenses. For the healthier group, health insurance played an important role in protecting against unexpected health problems and resulting economic risks.

Contrastingly, the impact of health insurance was not significant in the very healthy group. This may be because these respondents were in better health and faced lower health risks compared to the other groups; therefore, the impact of health insurance on their economic stability was limited. In addition, this group may prefer to rely on their own health management and preventive measures and have a relatively low need for health insurance.

## 4 Conclusion

Eradicating extreme poverty and preventing recurring poverty among rural households have been the focus of the Chinese Government’s poverty management efforts over the past two decades. Vulnerability to poverty is a typical characteristic of relative poverty and an important indicator of whether rural residents are likely to experience recurring poverty. Rural residents with at least one health insurance policy had a lower vulnerability to poverty than those without any health insurance coverage. Our findings indicated that health insurance coverage reduced vulnerability to poverty among rural residents in China. Our study contributes to the emerging literature on this topic by providing comprehensive nationwide empirical evidence and examining differences in the effect of health insurance based on having a second health insurance policy and by age and income. Our results directly inform estimates of the long-term effects of health insurance subsidies and social policies for rural residents.

We could not precisely determine the exact channel through which vulnerability to poverty was reduced. Owing to the episodic and unpredictable nature of healthcare expenditures, investing in health insurance may increase short-term financial burdens. However, health insurance can provide an important economic safety net that mitigates potential healthcare spending risks in the long term. Thus, even if some households do not receive direct health insurance benefits in the short term, they benefit because health insurance reduces their financial risk when faced with major medical expenses. In addition, health insurance may influence households’ health behaviors and healthcare consumption habits, promoting a focus on preventive care, thus reducing future healthcare expenditures.

This study makes several contributions. First, this study adopted the most comprehensive and up-to-date micro-survey data to identify the causal relationship between rural health insurance and vulnerability to poverty. This study used the entire period of health insurance implementation and continuous tracking surveys, providing the latest empirical evidence for the comprehensive consolidation of the results of the poverty elimination policy. This study provided a scientific basis for preventing recurring poverty among rural residents. By comprehensively analyzing microdata, this study identified the mechanism of health insurance in various contexts, providing policymakers with powerful decision support. Further, this study focused on rural residents and examined the dynamic impact of having a second health insurance policy and the differences by age and income. This study broadened research on vulnerability to poverty and lengthened the assessment chain of health insurance. In addition, this study analyzed the long-term impact of rural health insurance on the economic stability of rural residents. By analyzing long-term data, this study revealed not only direct short-term effect of health insurance but also its indirect long-term effects. This provides an empirical basis for improving rural health insurance promotion and subsidy policies and could help policymakers design and implement targeted measures to improve the coverage and effectiveness of policies.

The significance of this study is threefold. First, it highlights the rural–urban disparity in health insurance—with 56% of the global rural population lacking coverage, which is much higher than the 22% in urban areas. The current study provides a case study for preventing rural residents in China from falling back into poverty owing to medical problems, and it provides policy references and empirical evidence for other countries to use for poverty reduction. Second, since 2023, the Chinese government has benefited more than 180 million rural low-income people seeking medical treatment by reducing the burden of medical costs by 188.35 billion yuan. Finally, this study can promote social equity by safeguarding rural residents’ medical rights and improving the quality of the medical service system, contributing to balanced socio-economic development.

## Data Availability

The datasets presented in this study can be found in online repositories. The names of the repository/repositories and accession number(s) can be found at: http://isss.pku.edu.cn/cfps/index.htm.

## Footnotes

^1^Absolute poverty refers to a condition where individuals or households cannot obtain basic necessities like food, clothing, and shelter, typically measured by a specific poverty line. Relative poverty is based on a society’s overall income level, where individuals or households have significantly lower incomes compared to the societal average, making it difficult for them to maintain a standard of living comparable to others. Regional poverty focuses on poverty within specific geographic areas, often reflecting issues caused by regional developmental imbalances or environmental factors. Poverty lines established by various countries and international organizations are based on “absolute poverty,” aiming to meet the minimum survival needs.

^2^Download the raw data at http://isss.pku.edu.cn/cfps/index.htm.

^3^Available from http://isss.pku.edu.cn/cfps/index.htm.

^4^Income is the amount remaining after accounting for all wages, bonuses, cash benefits, and in-kind subsidies, after taxes have been deducted. Home equity is the net value of a house after subtracting housing-related debts from its market value. We chose this variable because 80% of Chinese residents’ assets are in housing, with an even higher proportion among rural residents.

^5^To ensure the reliability of the results, we selected the annual average exchange rates of RMB and USD for 2014, 2016, 2018, and 2020 to discount the poverty line.

^6^The CFPS database includes public, urban workers’, urban residents’, and new rural cooperative health insurance types.
